# An unusual complication of pyloric ring obstruction caused by flange of lumen apposing metal stent in endoscopic ultrasound-guided gallbladder drainage: A case report

**DOI:** 10.1097/MD.0000000000021017

**Published:** 2020-07-02

**Authors:** Seung Young Seo, Chang Hun Lee, In Hee Kim, Sang Wook Kim, Seung Ok Lee, Soo Teik Lee, Seong-Hun Kim

**Affiliations:** Department of Internal Medicine, Research Institute of Clinical Medicine of Jeonbuk National University-Biomedical Research Institute of Jeonbuk National University Hospital, Jeonju, Republic of Korea.

**Keywords:** acute cholecystitis, complication, endoscopic ultrasound-guided gallbladder drainage, gastric outlet obstruction, lumen-apposing metal stent

## Abstract

**Introduction:**

Endoscopic ultrasound-guided gallbladder drainage (EUS-GBD) is an alternative treatment option for patients with acute cholecystitis, especially for those who are unsuitable for cholecystectomy. Recently used luminal apposing metal stents (LAMS) in EUS-GBD has several advantages over standard metal stents. However, there is no current guideline on where to locate the LAMS when transgastric approach is required. This study reports a case of gastric outlet obstruction (GOO) by placing LAMS too close to the pyloric ring.

**Patient concerns:**

A 79-year-old female patient was referred to our department for evaluation of a large hepatic mass on abdominal ultrasound. Abdominal pain on right upper quadrant and spiking fever up to 38 °C appeared after liver biopsy. Abdominal ultrasound showed thickened GB wall and positive sonographic Murphy sign.

**Diagnoses:**

Intrahepatic cholangiocarcinoma with multiple lung and intrahepatic metastasis, acute cholecystitis, and pyloric ring obstruction caused by flange of LAMS in EUS-GBD.

**Interventions:**

EUS-GBD via transgastric approach was performed with LAMS. After complete deployment of stent, esophagogastroduodenoscopy showed complete GOO by flange of LAMS. A gastroduodenal metal stent was inserted to relieve the GOO.

**Outcomes:**

The patient recovered well. She did not complain about obstruction induced symptom such as vomiting or abdominal fullness after gastroduodenal stent insertion.

**Conclusion:**

To the best of our knowledge, this is the first case report of EUS-GBD induced GOO. If physicians use LAMS as a transgastric approach in EUS-GBD, the puncture site should be carefully selected considering the size of the flange.

## 1 Introduction

Cholecystectomy is the treatment of choice for acute cholecystitis.^[1]^ However, some patients are unsuitable for surgery because of advanced age and/or multiple comorbidities. Percutaneous transhepatic gallbladder drainage (PTGBD) and endoscopic ultrasound-guided gallbladder drainage (EUS-GBD) are alternative treatment options for acute cholecystitis, especially for those who are unsuitable for cholecystectomy. EUS-GBD has several advantages over PTGBD such as absence of external drainage tube and widespread indication in patients with coagulopathy or ascites. However, EUS-GBD can result in several complications such as gastrointestinal bleeding and stent migration.^[2]^ Recently, physicians have developed various types of stents to overcome shortcomings of standard metal stents. In particular, lumen-apposing metal stent (LAMS) has a large flange which can reduce stent migration and leakage by attaching a lumen. However, disadvantages of LAMS's flange are not exactly known.^[3]^ Here in, we present a unique case of gastric outlet obstruction (GOO) by placing the LAMS too close to the pyloric ring in a patient with advanced intrahepatic cholangiocarcinoma and acute cholecystitis. This case report was approved by the Institutional Review Board of Jeonbuk National University Hospital (IRB No. 2019-08-004), and the patient has signed informed consent to publication of the case.

## 2 Case report

**Figure 1:**
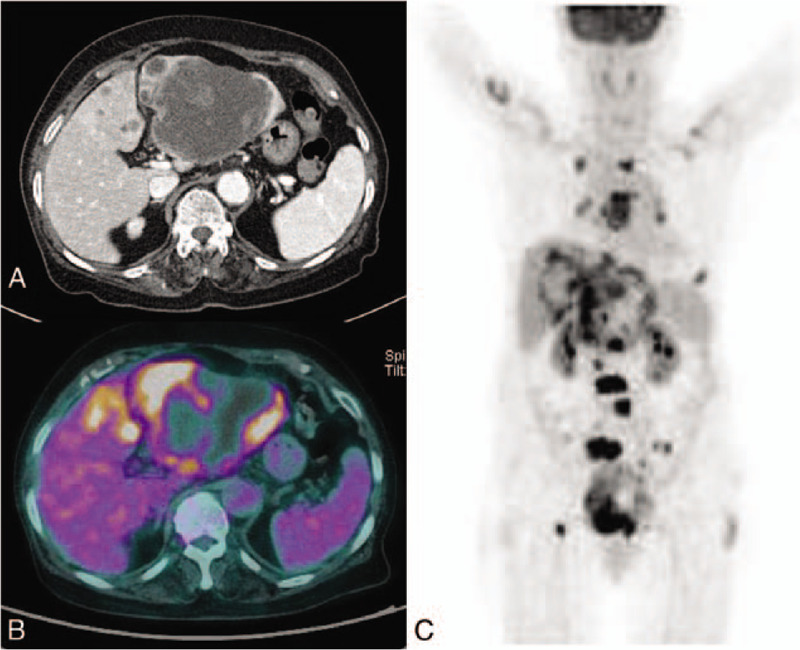
Abdominal computed tomography (CT) and Positron emission tomography-computed tomography (PET-CT). (A) About 10 cm sized, relatively well-defined hepatic mass with lobulated margin and peripheral rim enhancement. Multiple variable sized hepatic mass lesions showing similar characteristic were detected at left lobe. (B) and (C) ^18^F-fluorodeoxyglucose (FDG)-avid malignancy in left lobe of liver with multiple intrahepatic metastasis. Multiple lung, bone, right pleural metastasis, and peritoneal carcinomatosis were also shown.

A 79-year-old female patient who suffered epigastric pain without fever of 4 weeks in duration was referred to our department from primary health care unit because of a large hepatic mass on abdominal ultrasound. She had no prior pathological condition except hypertension. She also complained of general weakness and weight loss of 7 kg recently. In physical examination, the abdomen was soft and flat. There was no definitive direct tenderness. Laboratory findings showed mild elevation of alkaline phosphatase (224 IU/L) and gamma-glutamyltransferase (256 IU/L). Serum carbohydrate antigen 19-9 (CA19-9) level was 13233.13 U/mL (normal range: 0–37 U/mL). Abdominal enhanced computed tomography (CT) revealed about 10 cm sized, relatively well-defined hepatic mass with lobulated margin and peripheral rim enhancement at left lobe. Multiple variable sized hepatic mass lesions showing similar characteristic were detected at the left lobe (Fig. 1A). About 1 cm sized round shaped enhancing nodules were noted at the posterior side of the right psoas muscle and right buttock subcutaneous layer. Positron emission tomography-computed tomography (PET-CT) showed ^18^F-fluorodeoxyglucose (FDG)-avid malignancy in the left lobe of liver with multiple intrahepatic metastasis. Multiple lung, bone, right pleural metastasis, and peritoneal carcinomatosis were also shown on PET-CT (Fig. 1B and C). For histologic diagnosis, liver biopsy was performed. The biopsy result was adenocarcinoma favor cholangiocarcinoma. She was diagnosed with advanced intrahepatic cholangiocarcinoma. The next day of liver biopsy, she complained of abdominal pain on the right upper quadrant and spiking fever up to 39.1 °C. Direct tenderness on right upper quadrant and positive Murphy sign were revealed on physical examination. Laboratory findings showed leukocytosis (16.16 × 10^3^/μL) and elevated high-sensitivity C-reactive protein (157.26 mg/L). Abdominal sonography was performed and acute cholecystitis with sludge was established.

**Figure 2:**
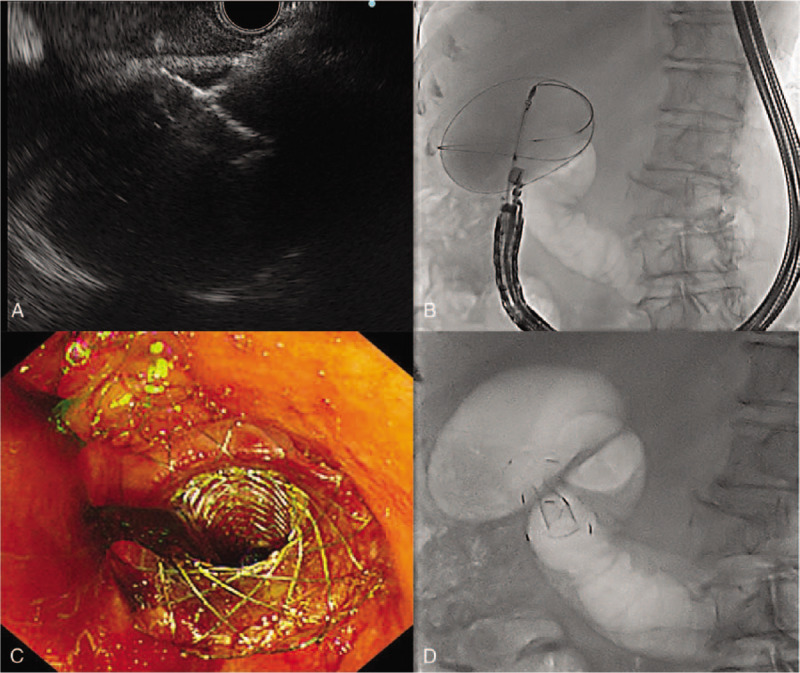
EUS-GBD using LAMS. (A) and (B) Puncturing GB and guidewire insertion. (C) and (D) After complete deployment of LAMS. EUS = endoscopic ultrasound; GBD = gallbladder drainage; LAMS = lumen-apposing metal stent.

**Figure 3:**
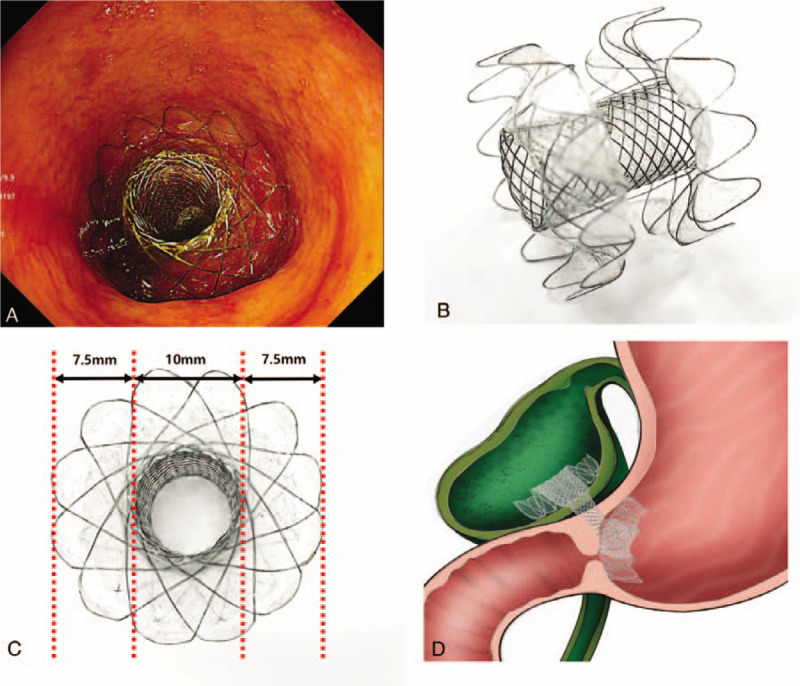
Complete gastric outlet obstruction by the flange of LAMS. (A) Complete gastric outlet obstruction by the flange of LAMS. (B) and (C) LAMS (20 mm in length, 10 mm in diameter, and 7.5 mm wide flange, LAMS, Hot-Spaxus, Taewoong Medical). (D) Schematic illustration of pyloric ring obstruction caused by the flange of LAMS. LAMS = lumen-apposing metal stent.

**Figure 4:**
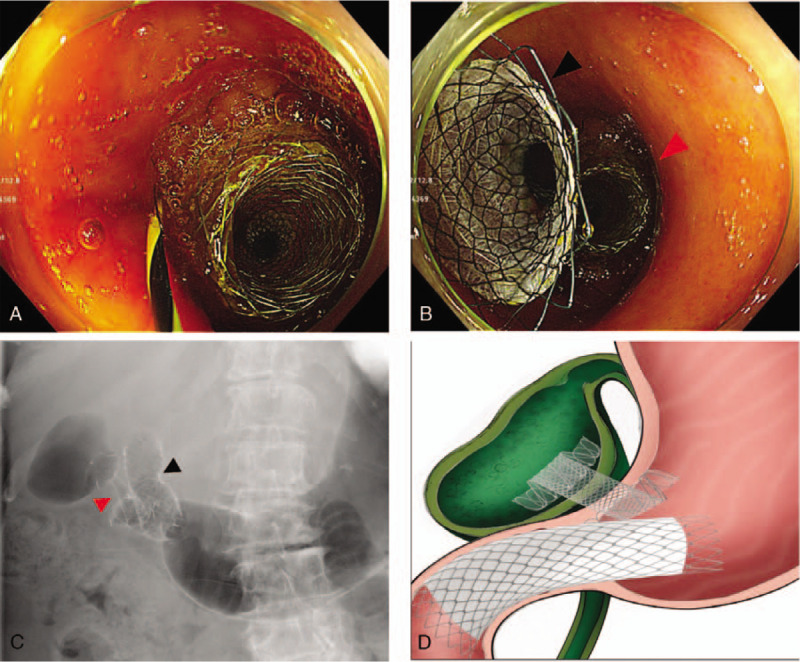
(A)–(C) A gastroduodenal metal stent insertion. (D) Schematic illustration of EUS-GBD using LAMS and gastroduodenal metal stent insertion for relieving gastric outlet obstruction. Red arrow: LAMS, Black arrow: gastroduodenal metal stent. EUS = endoscopic ultrasound; GBD = gallbladder drainage; LAMS = lumen-apposing metal stent.

We recommend PTGBD because cholecystectomy would be less advantageous. However, she rejected external drainage. Therefore, EUS-GBD was performed using linear array echoendoscope (GF-UCT260, EU-ME 2, Olympus) and LAMS (20 mm in length, 10 mm in diameter, and 7.5 mm wide flange, LAMS, Hot-Spaxus, Taewoong Medical) under conscious sedation. We first planned a transduodenal approach. However, there was deformity of duodenum bulb due to liver mass. Furthermore, a safe puncture route of transduodenal approach could not be secured due to collateral vessels around GB. Thus, we punctured GB with a 19-gauge FNA needle (EchoTip, Cook medical, Winston-Salem, NC, USA) via transgastric approach. A 0.025-in. guidewire (VisiGlide; Olympus Medical Systems, Tokyo, Japan) was inserted through the FNA needle into the GB and the LAMS was inserted securely (Fig. 2). After complete deployment of the stent, a large amount of dark brown colored fluid and sludge were drained via the LAMS. However, we could not observe the pyloric ring in the oblique view. Thus, we changed to esophagogastroduodenoscopy (EGD) to observe the position of LAMS. A complete gastric outlet obstruction by the flange of LAMS was noted on EGD (Fig. 3). We attempted to observe the pyloric ring by pushing the flange with a cap on the EGD. The puncture position of the LAMS was very close to the pyloric ring and the flange was closing the pyloric ring very firmly. We convinced that LAMS would cause gastric outlet obstruction. However, we could not eliminate LAMS. Thus, we decided to insert the gastroduodenal stent immediately after discussing her short life expectancy and the risk of repetitive procedures. To relieve the gastric outlet obstruction, a gastroduodenal metal stent (60 mm in length, 20 mm in diameter, partial covered, Taewoong Medical, Gimpo, Korea) was inserted (Fig. 4). Next morning, she started soft meal. She did not show any gastric outlet obstruction sign such as vomiting or abdominal fullness. Her abdominal pain and signs of inflammation such as fever and laboratory findings also improved. She refused palliative chemotherapy. She was discharged from hospital 3 days later.

## 3 Discussion

EUS-GBD is relatively new in the field of therapeutic EUS for internal drainage by creating a fistula between GB and gastrointestinal tract. It is an alternative treatment option for patients with acute cholecystitis, especially for those who are unsuitable for cholecystectomy. Most studies about EUS-GBD have enrolled patients with acute cholecystitis who are poor surgical candidates or who refuse to undergo PTGBD. PTGBD is another alternative treatment option for acute cholecystitis. However, as mentioned above it, PTGBD has several disadvantages over EUS-GBD such as presence of external drainage tube and relative contraindications in patients with coagulopathy or ascites. Also, PTGBD can cause postoperative pain in about 12% of patients^[4]^ and multiple additional interventions might be needed for catheter obstruction or migration. Furthermore, a recent study has reported that patients who undergo PTGBD show a tendency to remain poor surgical candidates during follow-up and require internal drainage using EUS-GBD.^[5]^ Regarding these limitations of PTGBD, EUS-GBD is an attractive treatment option for patients with acute cholecystitis who are poor surgical candidates.^[6]^ In this case, she underwent EUS-GBD because of her preference for EUS-GBD rather than PTGBD, her advanced old age, and metastatic intrahepatic cholangiocarcinoma.

The site of puncture when performing EUS-GBD is transgastric or transduodenal. The decision of puncture site depends on endoscopist's preference after assessing the patient's anatomical site of maximal direct apposition between GB and gastrointestinal tract. We performed a transgastric approach in this case because we could not confirm a safe route for transduodenal approach due to duodenal deformity and collateral vessels around the GB. After deciding the puncture site, EUS-GBD can be performed using plastic stent or self-expandable metal stent (SEMS). Several pooled analyses about the safety and efficacy of EUS-GBD have reported that technical and clinical success rates are as high as 97% and 99%, respectively, when including all stent types (plastic, SEMS, and LAMS).^[4,7–9]^ High risk of complications such as early occlusion of plastic stents and stent migration of covered SEMS that could result in bile leakage induced peritonitis are limitations of EUS-GBD.^[10]^ To overcome these limitations, LAMS, a saddle-shaped stent with double-walled flanges perpendicular to the lumen on both sides, has been developed. A recent systematic review reported that stent migration of EUS-GBD using LAMS occurred in only 2 cases among 183 patients.^[3]^ However, gastric outlet obstruction due to LAMS has not been reported so far. In the present case, EUS-GBD using LAMS was performed. Generally known complications such as stent migration, bile leakage, or bleeding did not occur. However, after complete deployment of stent, esophagogastroduodenoscopy showed complete gastric outlet obstruction by flange of LAMS. Thus, a gastroduodenal metal stent was inserted to relieve the gastric outlet obstruction. To the best of our knowledge, this is the first case report of EUS-GBD induced gastric outlet obstruction. LAMS has a saddle-shaped flange to overcome disadvantages of conventional metal stents. LAMS also has increased width of the flange and shortening force for lumen apposing of bilateral flanges to reduce migration and bile leakage of the stent. Due to its advanced features, flange can also block normal surrounding structures. We did not consider characteristics of this flange when puncturing an FNA needle with a transgastric route. Thus, the distance between the puncture site and the pyloric ring was smaller than the width of the flange. Thus, the expanded LAMS blocked the pyloric ring. The strong shortening of the bilateral flange also made it less likely for food to pass by the LAMS flange. Therefore, our patient underwent a gastroduodenal metal stent for gastric outlet obstruction. She did not show any gastric outlet obstruction sign such as vomiting or abdominal fullness after discharge. The force of the LAMS flange to maintain its shape might have prevented migration of the gastroduodenal stent.

In conclusion, we report the first case of GOO by placing LAMS too close to the pyloric ring in a patient with advanced intrahepatic cholangiocarcinoma and acute cholecystitis. EUS-GBD is attractive treatment option in patients with acute cholecystitis who are unsuitable for cholecystectomy. The puncture site decision is made after assessing the patient's anatomy maximal direct apposition between GB and gastrointestinal tract. However, the physician must consider the size of a flange of the LAMS so that the LAMS does not block the pyloric ring when puncturing the needle with a transgastric approach due to inherent characteristics of LAMS flange. Therefore, we recommend the puncture site must be placed at least 1 cm apart from the pyloric ring in case of a transgastric approach.

## Author contributions

**Investigation:** Seung Young Seo, Chang Hun Lee, In Hee Kim, Seong-Hun Kim.

**Visualization:** Sang Wook Kim, Seung Ok Lee, Seong-Hun Kim.

**Writing – original draft:** Seung Young Seo

**Writing – review & editing:** Seung Young Seo, Soo Teik Lee, Seong-Hun Kim.
